# Comparison of clinical outcomes of frozen-thawed D5 and D6 blastocysts undergoing preimplantation genetic testing

**DOI:** 10.1186/s12967-022-03762-4

**Published:** 2022-11-26

**Authors:** Guanling Yu, Shuiying Ma, Hui Liu, Yujin Liu, Haozhen Zhang, Wenjia Zhang, Keliang Wu

**Affiliations:** 1 Center for Reproductive Medicine, Shandong University, Jinan, 250012 Shandong China; 2 Department of Anesthesiology, Shandong Provincial Hospital Affiliated to Shandong First Medical University, Jinan, 250021 Shandong China

**Keywords:** Frozen-thawed blastocyst, Embryo transfer, Assisted reproduction, Preimplantation genetic testing (PGT)

## Abstract

**Background:**

This study aimed to analyze the clinical outcomes of blastocyst which undergo the preimplantation genetic testing (PGT) transplantation from frozen-thawed D5 and D6. In addition, the effect of blastocyst grade on clinical and neonatal outcomes was also investigated in this study.

**Methods:**

The pregnancy and miscarriage rates of 1130 cycles of frozen embryo transfer, including 784 D5 frozen embryos and 346 D6 frozen embryos in the Reproductive Hospital of Shandong University from January to December 2020 were analyzed. Gardner blastocyst scoring was used for blastocyst evaluation.

**Results:**

The pregnancy rate of D5 blastocyst was significantly higher, whereas the miscarriage rate of D5 blastocyst was lower, than that of D6 blastocyst tissue biopsy. No significant difference was observed in birth weight and low birth weight of D5 blastocyst and D6 blastocyst, preterm birth, gestational age, and neonatal sex. Frozen-thawed D5 blastocysts have higher pregnancy success rates and lower miscarriage rates compared to D6 blastocysts.

**Conclusion:**

Therefore, both blastocyst grade and embryo biopsy date must be considered when transferring frozen embryos.

**Supplementary Information:**

The online version contains supplementary material available at 10.1186/s12967-022-03762-4.

## Introduction

Preimplantation genetic testing (PGT) is an important step in the process of assisted reproduction that affects pregnancy outcomes [1]. PGT has been employed as an embryo selection tool for patients undergoing in vitro fertilization (IVF) to ensure higher implantation rates or at least shorter gestational times [2]. PGT is an organic combination of assisted reproductive technology and modern molecular genetic diagnosis technology, and is an important part of preventive medicine [3]. Compared with prenatal diagnosis, the superiority of PGT lies in the screening and diagnosis of preimplantation embryos and the realization of eugenics from the source of pregnancy [1]. The vitrification technique post-PGT greatly shortens the operation time and reduces the physical damage to the blastocyst by trophoblast sampling during the PGT [4]. However, the impact of PGT sampling at different developmental times of blastocysts on embryonic developmental outcomes remains unclear.

With the wide application of blastocyst culture and blastocyst vitrification freeze-thaw technology in the field of reproductive medicine, the proportion of frozen-thawed embryo transfer after whole embryo freezing has increased significantly [5]. Frozen embryo transfer technology can reduce maternal discomfort to freshly transferred embryos and improve the synchrony between embryos and endometrium under the stress of promoting ovulation [6]. It has been reported that patients with high risk for ovarian hyperstimulation syndrome, endometrial abnormalities, hyper-progesterone, and systemic diseases who are not candidates for fresh transfer have a significantly reduced risk of frozen embryo transfer [7]. It has also been reported that clinical pregnancy outcomes after frozen-thawed embryo transfer are better than those with fresh-cycle transfer [8].

The developmental days and quality of embryos are the key factors to determine whether frozen-thawed blastocyst transfer can lead to clinical pregnancy [9]. Due to hormonal priming of endometrial receptivity, programmed thawed blastocyst transfer cycles should have the same clinical outcome when 5th day (D5) or 6th day (D6) blastocysts are transferred for better endometrial-embryo synchronization [10]. However, PGT requires freeze-thaw sampling of frozen blastocysts prior to embryo transfer [11]. Therefore, trophoblast biopsies for D5 frozen-thawed blastocysts and D6 frozen-thawed blastocysts may have different effects on embryo implantation and development [12].

We compared the clinical outcomes of blastocyst transplantation post PGT at frozen-thawed D5 and frozen-thawed D6 in this study. This study aims to provide theoretical support for the establishment of a standardized operation procedure for frozen-thawed blastocyst sampling in PGT to enhance the clinical pregnancy outcomes to the greatest extent.

## Methods

### Study design

This study conducted research statistics on all patients who underwent frozen embryo transfer in the Reproductive Hospital of Shandong University from January to December 2020. There were 1130 cycles of frozen embryo transfer, including 784 D5 frozen embryos and 346 D6 frozen embryos. In frozen embryo transfer, COOK reagent was used to thaw embryos, and all embryos were thawed and cultured for 4.5–5.5 h before transplantation. The study was approved by Center for Reproductive Medicine, Shandong University.

### Study subjects

Inclusion criteria: aged between 18 and 45; infertility due to pelvic tubal factors or male factors; signed informed consent; requesting third-generation IVF and PGT due to a variety of reasons, such as multiple recurrent miscarriages, a family history of the disease, Chromosome abnormality, or the presence of a single-gene disease in one of their parents.

### Acquisition and culture of blastocysts

The ovulation induction protocols adopted include long-term protocol, short-term protocol, antagonist protocol, and micro-stimulation protocol. The oocytes were retrieved 36 h after the injection of human chorionic gonadotropin (hCG), and the obtained oocytes were placed in the fertilization medium. The semen was collected by masturbation or puncture, and the semen was processed by gradient centrifugation. All patients underwent intracytoplasmic sperm injection (ICSI) 39–41 h after injection of human chorionic gonadotropin (hCG), and then the fertilized eggs were transferred to the cleavage stage medium and cultured in a 37 °C incubator with 6% CO_2_. 16–20 h after insemination, the formation of pronuclei was observed, and abnormal fertilization embryos were picked out, and the rest were cultured for 48 h. Embryos on the third day were observed, and the quality of cleavage stage embryos was evaluated. Embryos were transferred to blastocyst stage medium and cultured to D5 or D6 blastocysts.

### Blastocyst scoring

The blastocysts were scored by Gardner blastocyst scoring method, and those with D5 score ≥ 4AA, 4AB, 4BA, 4BB or D6 score ≥ 4AA, 4AB, 4BA, 4BB were determined as high-quality blastocysts.

### Vitrification of blastocysts

The blastocysts were laser drilled using Hamilton software. The blastocyst inner cell mass should be avoided when laser drilling. The blastocyst shrank about 5 min after the blasting fluid flowed out. The blastocysts were transferred to the equilibration solution for 2 min at 37 °C temperature. Blastocysts were transferred to vitrification solution for no more than 30 s. Blastocysts were placed on labeled cryo-carriers and quickly immersed in liquid nitrogen for preservation.

### Blastocyst thawing

The whole process of embryo thawing was performed at 37 °C using Cook Thawing Solution. Frozen carrier rods were immersed in thawing solution for no more than 2 min. Embryos were quickly moved to Wash Buffer for 3 min and then move to Base fluid for 5 min. Finally, the embryos were transferred to blastocyst medium and cultured at 37 °C in a plate incubator. The blastocyst survival was observed after 2 h of recovery.

### Blastocyst transfer

Endometrial preparation for blastocyst transfer is divided into natural cycle preparation and artificial cycle preparation. All frozen embryo cycle preparation protocols were treated with our center’s routine protocol. The patient’s embryos were thawed and transplanted 4–5 h later. All procedures were performed by the same doctor on the same day. At the time of embryo transfer, the recovery of embryo was observed by laboratory experimenter and the time of culture was recorded.

### Post-embryo transfer management and pregnancy outcome judgment

The patient was discharged after 40 min of supine rest, and continued luteal support therapy under the guidance of the doctor. Biochemical pregnancy: 12 days after transplantation, the blood was positive for hCG; clinical pregnancy: 30 days after transplantation, the number of gestational sacs in the uterine cavity and the presence or absence of cardiac pulsation were examined by vaginal B-ultrasound. Clinical pregnancy rate = number of clinical pregnancy cycles/number of transplant cycles ⨯ 100%; embryo implantation rate = number of gestational sacs detected under B-ultrasound/number of blastocysts transferred ⨯ 100%; ectopic pregnancy rate = number of ectopic pregnancy cycles/The number of clinical pregnancy cycles × 100%; the rate of monozygotic twins = the number of monozygotic twin pregnancy cycles/the number of clinical pregnancy cycles × 100%.

### Statistical analysis

Data analysis was performed by SPSS 16.0 statistical software. Normally distributed measurement data was expressed as x ± s. independent samples t test was used for comparison between groups, and F test was used for comparison between multiple groups. Count data was expressed as %. The χ test was used for comparison between groups. P < 0.05 was considered as statistically significant.

## Results

### Characteristics of patients received blastocyst transplantation from frozen-thawed D5 and frozen-thawed D6 in the study

The characteristics of patients received blastocyst transplantation from frozen-thawed D5 and frozen-thawed D6 in the study were shown in Table 1. A total of 1130 frozen embryos were transferred, including 784 frozen embryos from D5 and 346 frozen embryos from D6. The rate of obtaining high-quality blastocysts in the D5 group was higher than that in the D6 group. The proportion of partial hatching and complete hatching in the D5 group was higher than that in the D6 group.

**Table 1:** Characteristics of patients received blastocyst transplantation from frozen-thawed D5 and frozen-thawed D6 in the study

Characteristics	Study group		p
	D5 (n = 782)	D6 (n = 343)	
Age (years)	32.5 ± 4.5	33.1 ± 4.7	0.050
Husband’s age (years)	33.3 ± 5.0	33.9 ± 4.8	0.035
Quality of blastocysts			
High	743 (95%)	251 (73.2%)	< 0.001
General	39 (5%)	92 (26.8%)	
Incubation outcomes			
Hatched	593 (75.8%)	228 (66.5%)	< 0.001
Full recovery	174 (22.3%)	90 (26.2%)	
Partial recovery	15 (1.9%)	25 (7.3%)	

Values were expressed as n (percentage, %) or mean ± SD. p values were derived from Mann Whitney test. Fisher’s exact test or Chi-square was used for assessing distribution of observations

### Blastocysts from frozen-thawed D5 and frozen-thawed D6

**Fig. 1:**
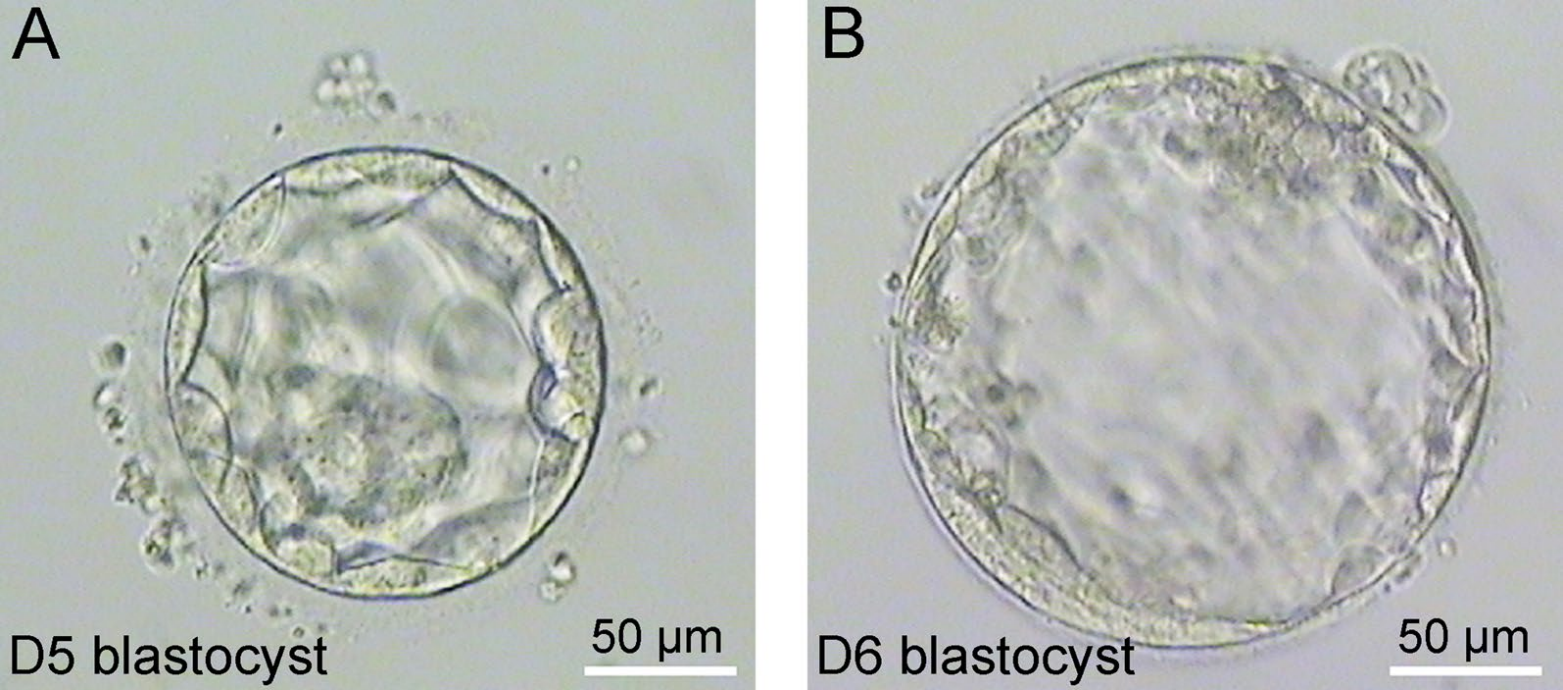
Representative images of blastocysts from frozen-thawed D5 (**A**) and frozen-thawed D6 (**B**)

Representative images of blastocysts from frozen-thawed D5 (Fig. 1A) and frozen-thawed D6 (Fig. 1B) were shown in Fig. 1 and Additional file 1: Fig. S1. Compared with D6 blastocysts, D5 blastocysts have smaller blastocoel cavity, fewer trophoblast cells, and larger overall cell size than D6 blastocysts. D5 trophoblast cells were more likely to rupture when aspirated, and the number and integrity of cells obtained at biopsy were worse than D6. The blastocysts images recorded at the time of biopsy were shown in Additional file 1: Fig. S2.

### The pregnancy ratio and abortion ratio between patients received blastocyst transplantation from frozen-thawed D5 and frozen-thawed D6

**Fig. 2:**
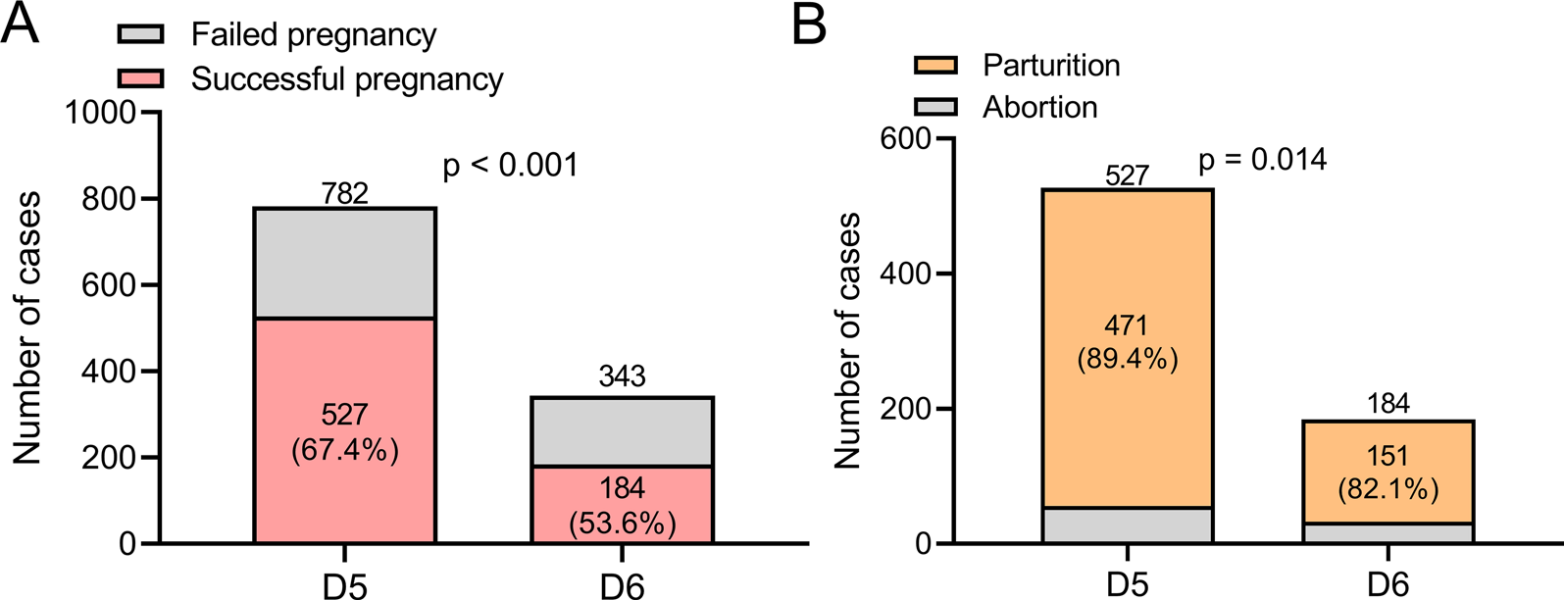
Comparisons of the pregnancy ratio (**A**) and abortion ratio (**B**) between patients received blastocyst transplantation from frozen-thawed D5 and frozen-thawed D6. Fisher’s exact test

The outcomes of frozen-thawed D5 and frozen-thawed D6 blastocysts transplantation were further analyzed. As shown in Fig. 2A, the pregnancy rate of D6 blastocyst transfer was remarkably lower than that of D5 group. Consistently, the abortion rate of D5 blastocyst transfer was significantly lower than that of D6 group (Fig. 2B).

### The pregnancy ratio and abortion ratio between patients received different quality of blastocyst transplantation from frozen-thawed D5 and frozen-thawed D6

**Fig. 3:**
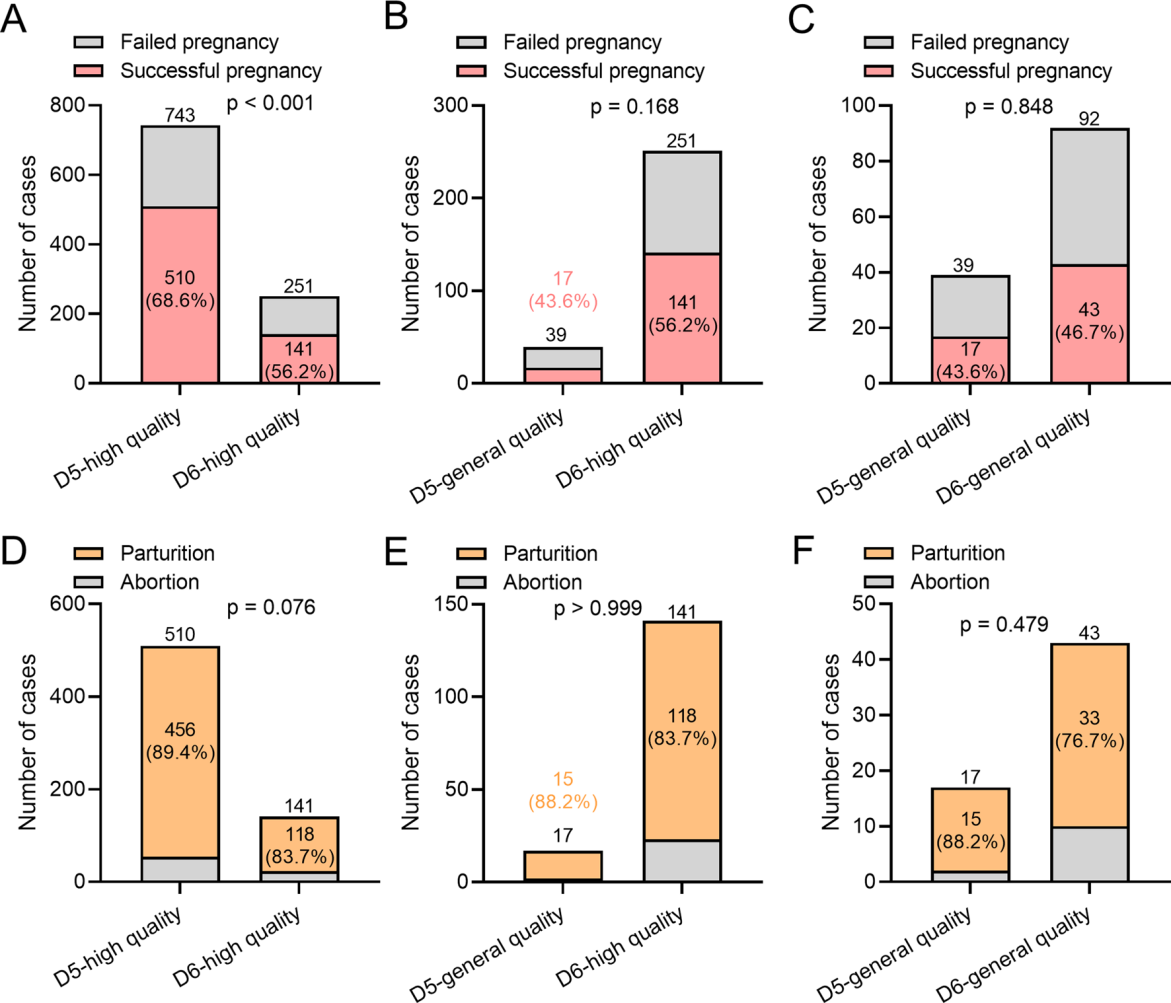
Comparisons of the pregnancy ratio (**A**-**C**) and abortion ratio (**D**-**F**) between patients received different quality of blastocyst transplantation from frozen-thawed D5 and frozen-thawed D6. Fisher’s exact test

The effects of different quality blastocysts D5 and D6 on miscarriage rate and post-transfer pregnancy rate were further investigated. We found that D5 high-quality blastocysts exhibited an enhanced pregnancy rate than D6 high-quality blastocysts, whereas the abortion rate between D5 high-quality blastocysts and D6 high-quality blastocysts were not significant difference (Fig. 3A, D). However, there were no differences in pregnancy and miscarriage rates between D5 normal quality blastocysts and D6 high quality blastocysts (Fig. 3B, E). Likewise, there were no differences in pregnancy and miscarriage rates between D5 average quality blastocysts and D6 average quality blastocysts (Fig. 3C, F). D5 high quality blastocyst transfer can improve pregnancy rate (Additional file 1: Fig. S3A) and reduce miscarriage rate (Additional file 1: Fig. S3B) relative to D6 normal quality blastocyst. On the other hand, we compared the effects of D5 blastocysts of different quality on pregnancy and miscarriage rates. High-quality blastocysts improved pregnancy rates (Additional file 1: Fig. S4A), but did not affect miscarriage rates (Additional file 1: Fig. S4B).

### The pregnancy ratio and abortion ratio between patients received different incubation outcomes of blastocyst transplantation from frozen-thawed D5 and frozen-thawed D6

**Fig. 4:**
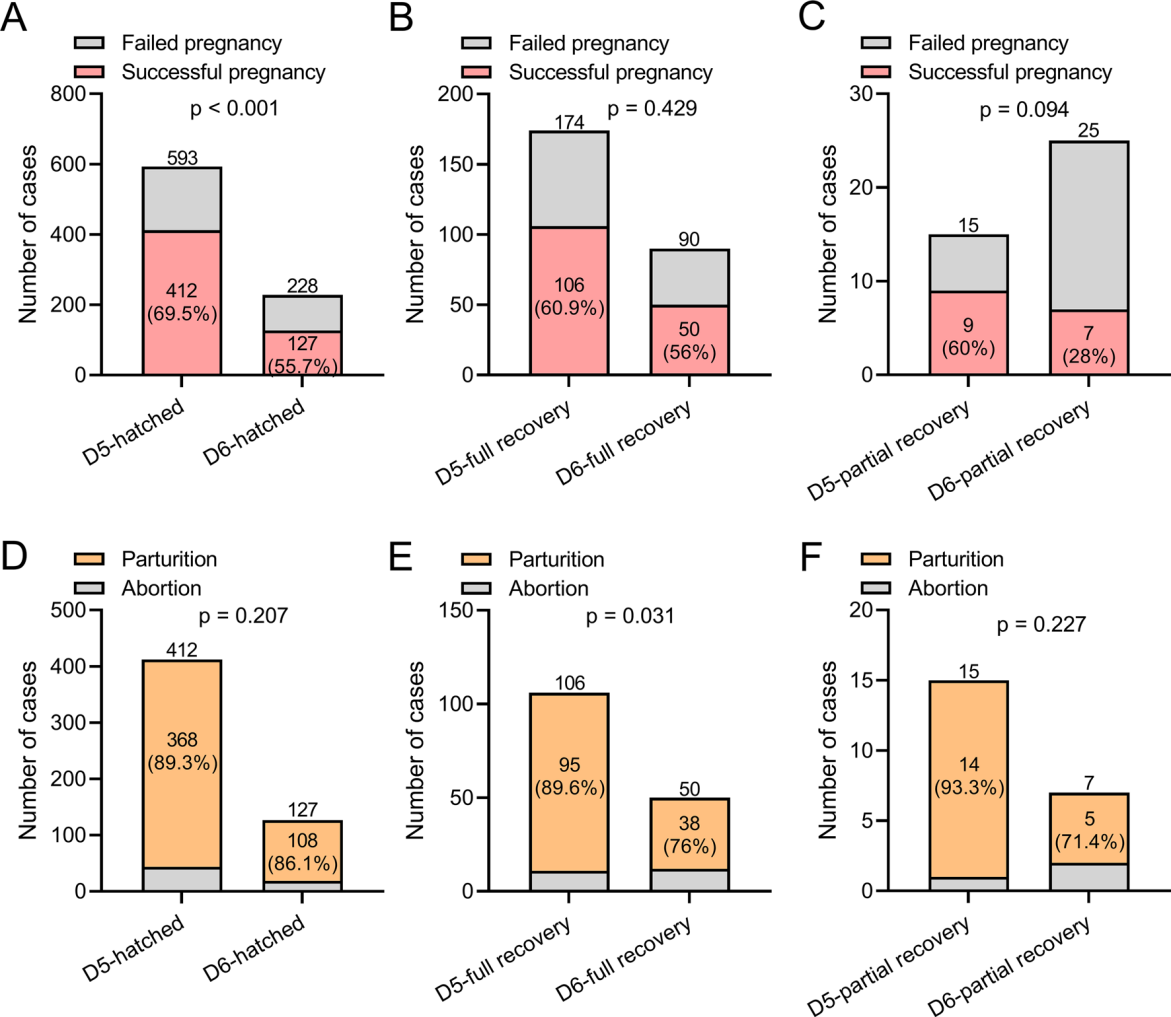
Comparisons of the pregnancy ratio (**A**-**C**) and abortion ratio (**D**-**F**) between patients received different incubation outcomes of blastocyst transplantation from frozen-thawed D5 and frozen-thawed D6. Fisher’s exact test

Comparisons of the pregnancy ratio and abortion ratio between patients received different incubation outcomes of blastocyst transplantation from frozen-thawed D5 and frozen-thawed D6 were shown in Fig. 4. D5 hatched blastocysts had a higher pregnancy rate than D6 hatched blastocysts (Fig. 4A), but there was no significant difference in the miscarriage rate between the two groups (Fig. 4D). D5 full recovery blastocysts had a higher pregnancy rate and lower miscarriage rate than D6 full recovery blastocysts, but only the miscarriage rate was significantly different (Fig. 4B, E). Similarly, D5 partially resuscitated blastocysts had a higher pregnancy rate and lower miscarriage rate than D6 partially resuscitated blastocysts, but there was no significant difference, which may be due to the small sample size (Fig. 4C, F). In addition, we also compared the differences in pregnancy and miscarriage rates between different recovery states of D5 blastocysts. D5 hatched blastocysts improved pregnancy rates compared to D5 fully resuscitated blastocysts, but had no effect on miscarriage rates (Additional file 1: Fig. S5A, B). D5 hatched blastocysts showed no difference in pregnancy and miscarriage rates compared to D5 partially resuscitated blastocysts (Additional file 1: Fig. S5C, D).

## Discussion

Chromosomal screening has become a routine test in many IVF practices around the world. Reliable information on the euploid status of embryos creates a solid basis for significantly improved outcomes of IVF treatment. PGT is suitable for recurrent miscarriage and the prevention of genetic diseases. The purpose of PGT is to block chromosomal diseases and single-gene genetic diseases before successful pregnancy, which can significantly increase the pregnancy rate and live birth rate. Previous FISH-based preimplantation genetic screening (PGS) has shown that more rapidly developing cleavage stage embryos are more likely to have various genetic abnormalities [13]. Although data on the timing of blastocyst development and the genetic status of embryos are limited and fragmented, several studies have reported higher clinical pregnancy rates in non-PGS cases in which faster developing blastocysts were selected for embryo transfer [14]. Previous studies have shown that embryos that pass cell cycle checkpoints without delay are more likely to rapidly complete mitosis and inflate or hatch blastocysts in the morning on day 5 (~ 116 h) [15]. The limitation of PGT is that it is an invasive test that requires the extraction of a portion of the embryo (biopsy of the embryo) for genetic testing. Although some studies have shown that embryo biopsy performed at D5 has no negative impact on embryo viability, inappropriate manipulation may still affect embryo survival and development. In addition, screening for PGT resulted in a substantial reduction in the number of available embryos. In many cases of PGT, the patient’s euploid embryos have similar morphological features, but the embryos can be biopsied (and subsequently frozen) at different times: most commonly on day 5 or 6, or even on day 7 [16]. To optimize the clinical outcome of a PGT cycle, another variable should be considered in addition to embryo morphology. This variable is the time a particular euploid embryo is available for biopsy [17]. The correlations between embryonic developmental dynamics, blastocyst chromosomal status, and clinical outcomes of the PGS cycle still require more studies to analyze.

Embryo quality, endometrial receptivity and synchronization between embryos and endometrium are the three most critical elements influencing the outcome of assisted reproductive pregnancy [18]. Embryo quality is the main determinant of pregnancy outcome [19, 20]. High-quality blastocysts with better developmental potential need to be evaluated in terms of morphology, developmental days, and preimplantation genetic diagnosis [21]. Developmental days are an important evaluation factor. At present, the results reported in the literature are inconsistent about whether the blastocyst development day exert influence on the implantation rate and pregnancy rate [22]. It has been reported that D5 and D6 blastocysts showed similar euploidy rate [23]. A meta-analysis of 15 resuscitated blastocyst transfer studies included 2502 frozen-thawed blastocyst transfer cases and found that the live birth rate, continued pregnancy rate, and clinical pregnancy rate after blastocyst transfer in the D6 group were remarkably lower than those in the D5 group. However, there was no statistical difference in the exact comparison of blastocyst transfer outcomes from D5 and D6 to the same stage [24]. Similar findings were also reported by Yang et al., who analyzed 1374 D5 blastocysts and 255 D6 blastocysts and found that D5 high-quality blastocysts and D6 high-quality blastocysts showed almost identical clinical pregnancy rate and implantation rate (52 0.4% vs. 52.6%; 38.9% vs. 35.6%) [25]. In addition, D4 embryos are not clinically used for PGT and embryo transfer because D4 embryos belong to the early stage of blastocyst development and cannot meet the requirements of PGT. While D7 blastocysts are in the metamorphic stage, and D7 blastocyst suppression resulted in significantly lower clinical pregnancy rates.

In fact, current mainstream research suggests that D5 embryos have better clinical outcomes than D3 and D6 embryos. The research of Kovalevsky et al. showed that the clinical pregnancy rate, implantation rate and baby carrier rate after freeze-thaw transfer of D5 blastocysts were significantly higher than those of D6 blastocysts [26]. It has been demonstrated that day 5 vitrified blastocysts have a higher clinical pregnancy rate than day 6 vitrified blastocysts [27]. In this study, we also reported that D5 blastocysts possessed enhanced transfer success rate and lower miscarriage rates relative to D6 blastocysts, which was also consistent with some previous studies. We reported that the high-quality blastocyst acquisition rate in D5 group was higher than that in D6 group, and the proportion of partial hatching and complete hatching in D5 group was higher than that in D6 group, with significant differences. During the biopsy, we also observed that D5 trophoblast cells were more prone to rupture upon aspiration, and the number and integrity of cells obtained were worse than those of D6. Therefore, D5 blastocysts yield less cell number and integrity than D6 blastocysts.

To investigate the impact of blastocyst development quality on pregnancy outcomes, we also compared the effects of D5 and D6 blastocysts of different quality on post-transfer pregnancy and miscarriage rates in this study. D5 high-quality blastocysts exhibited an enhanced pregnancy rate than D6 high-quality blastocysts, but there was no significant difference in the miscarriage rate between the two groups. There were no differences in pregnancy and miscarriage rates between D5 normal quality blastocysts and D6 high quality blastocysts. Likewise, no significant differences were observed in pregnancy and miscarriage rates between D5 average quality blastocysts and D6 average quality blastocysts. Our research suggests that the effect of embryo quality on D5 and D6 blastocysts is mainly reflected in the implantation success rate.

## Conclusion

In conclusion, we mainly compared the effects of embryo development time, embryo quality and different recovery status on the outcomes of frozen-thawed blastocyst transfer in this study. The present research shows that D5 blastocysts have higher embryo transfer success rates and lower miscarriage rates relative to D6 embryos. While D5 high-quality blastocysts had higher pregnancy rates than D6 high-quality blastocysts, no significant difference was observed in miscarriage rates between the two groups. Therefore, blastocyst grade and embryo biopsy date are two important factors that must be considered in clinical frozen-thawed blastocyst transfer.

## Supplementary Information

**Additional file 1: Figure S1.** More representative images of blastocysts from frozen-thawed D5 (A) and frozen-thawed D6 (B). **Figure S2.** Representative images of blastocysts from frozen-thawed D5 (A) and frozen-thawed D6 (B) during aspiration. **Figure S3.** Comparisons of the pregnancy ratio (A) and abortion ratio (B) between patients received high quality of blastocyst transplantation from frozen-thawed D5 and general quality from frozen-thawed D6. Fisher’s exact test. **Figure S4.** Comparisons of the pregnancy ratio (A) and abortion ratio (B) between patients received different quality of blastocyst transplantation from frozen-thawed D5. Fisher’s exact test. **Figure S5.** Comparisons of the pregnancy ratio (A, B) and abortion ratio (C, D) between patients received different incubation outcomes of blastocyst transplantation from frozen-thawed D5. Fisher’s exact test.

## Data Availability

All data generated or analysed during this study are included in this published article.

## Abbreviations

PGT: Preimplantation genetic testing
IVF: Vitro fertilization
hCG: Human chorionic gonadotropin
ICSI: Intracytoplasmic sperm injection
