# Supplementary material for: Comparison of clinical outcomes of frozen-thawed D5 and D6 blastocysts undergoing preimplantation genetic testing
Source: J Transl Med. 2022 Nov 26;20:545. doi: 10.1186/s12967-022-03762-4 (PMC9701440; doi:10.1186/s12967-022-03762-4)
Supplement: Supplementary file 1 — Additional file 1: Figure S1. More representative images of blastocysts from frozen-thawed D5 (A) and frozen-thawed D6 (B). Figure S2. Representative images of blastocysts from frozen-thawed D5 (A) and frozen-thawed D6 (B) during aspiration. Figure S3. Comparisons of the pregnancy ratio (A) and abortion ratio (B) between patients received high quality of blastocyst transplantation from frozen-thawed D5 and general quality from frozen-thawed D6. Fisher’s exact test. Figure S4. Comparisons of the pregnancy ratio (A) and abortion ratio (B) between patients received different quality of blastocyst transplantation from frozen-thawed D5. Fisher’s exact test. Figure S5. Comparisons of the pregnancy ratio (A, B) and abortion ratio (C, D) between patients received different incubation outcomes of blastocyst transplantation from frozen-thawed D5. Fisher’s exact test. [file 12967_2022_3762_MOESM1_ESM.docx]

**Additional file**


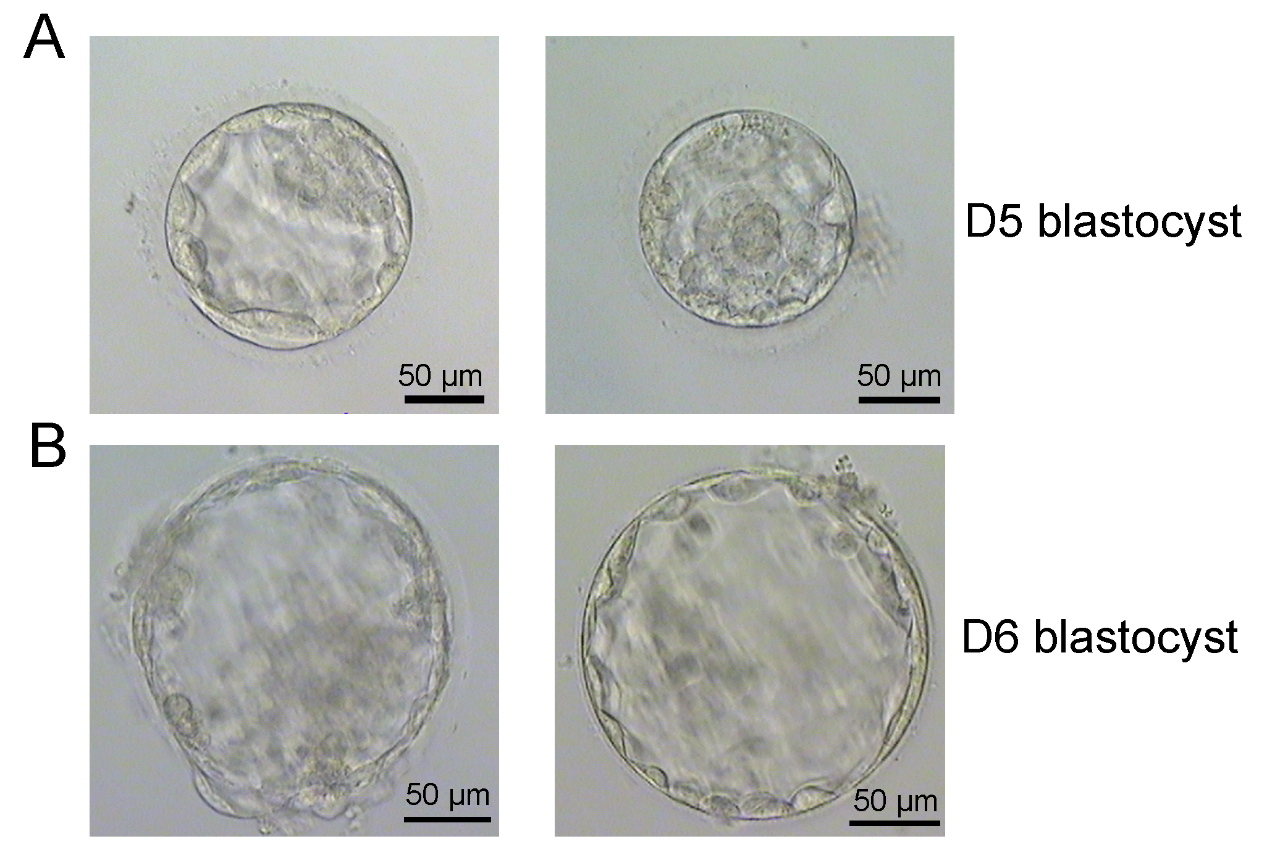


Figure S1. More representative images of blastocysts from frozen-thawed D5 (A) and frozen-thawed D6 (B).


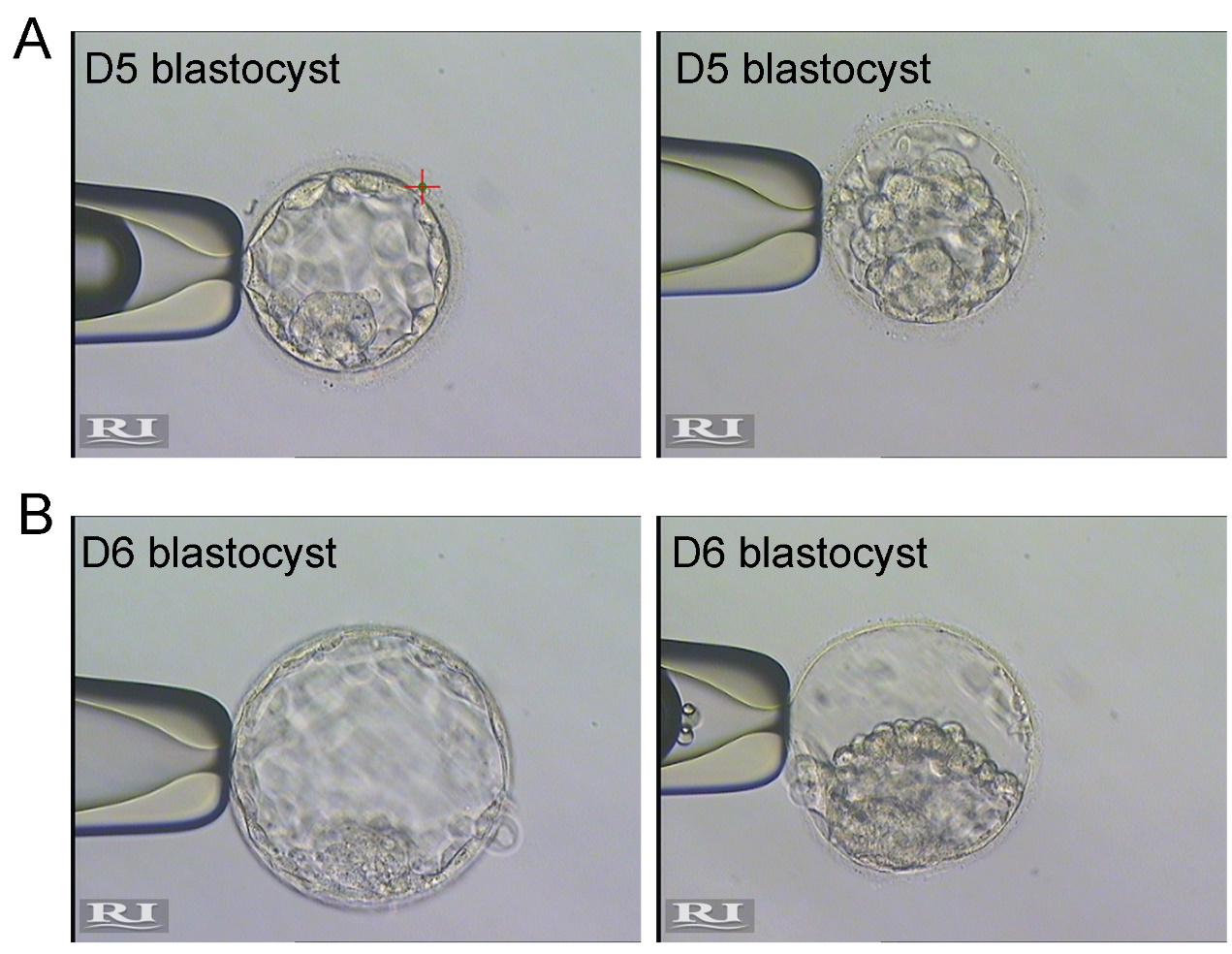


Figure S2. Representative images of blastocysts from frozen-thawed D5 (A) and frozen-thawed D6 (B) during aspiration.


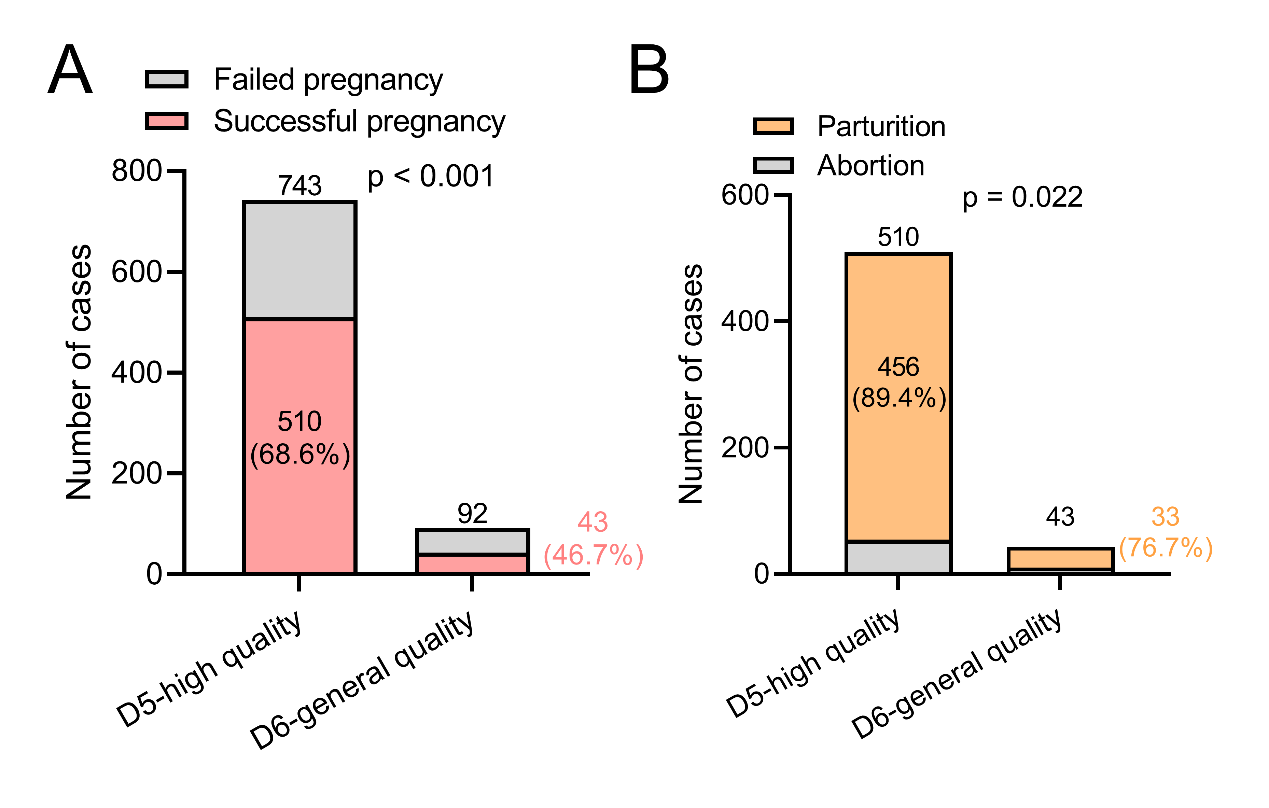


Figure S3. Comparisons of the pregnancy ratio (A) and abortion ratio (B) between patients received high quality of blastocyst transplantation from frozen-thawed D5 and general quality from frozen-thawed D6. Fisher’s exact test.


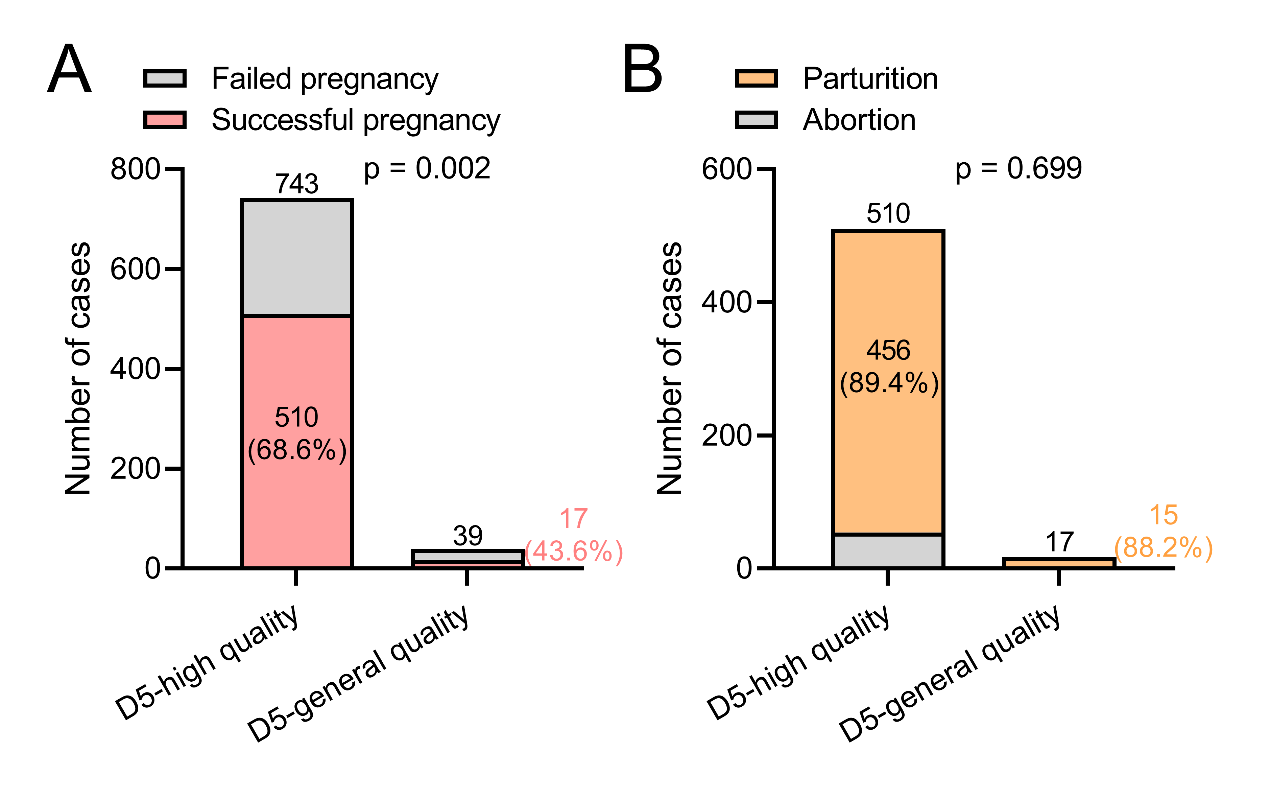


Figure S4. Comparisons of the pregnancy ratio (A) and abortion ratio (B) between patients received different quality of blastocyst transplantation from frozen-thawed D5. Fisher’s exact test.


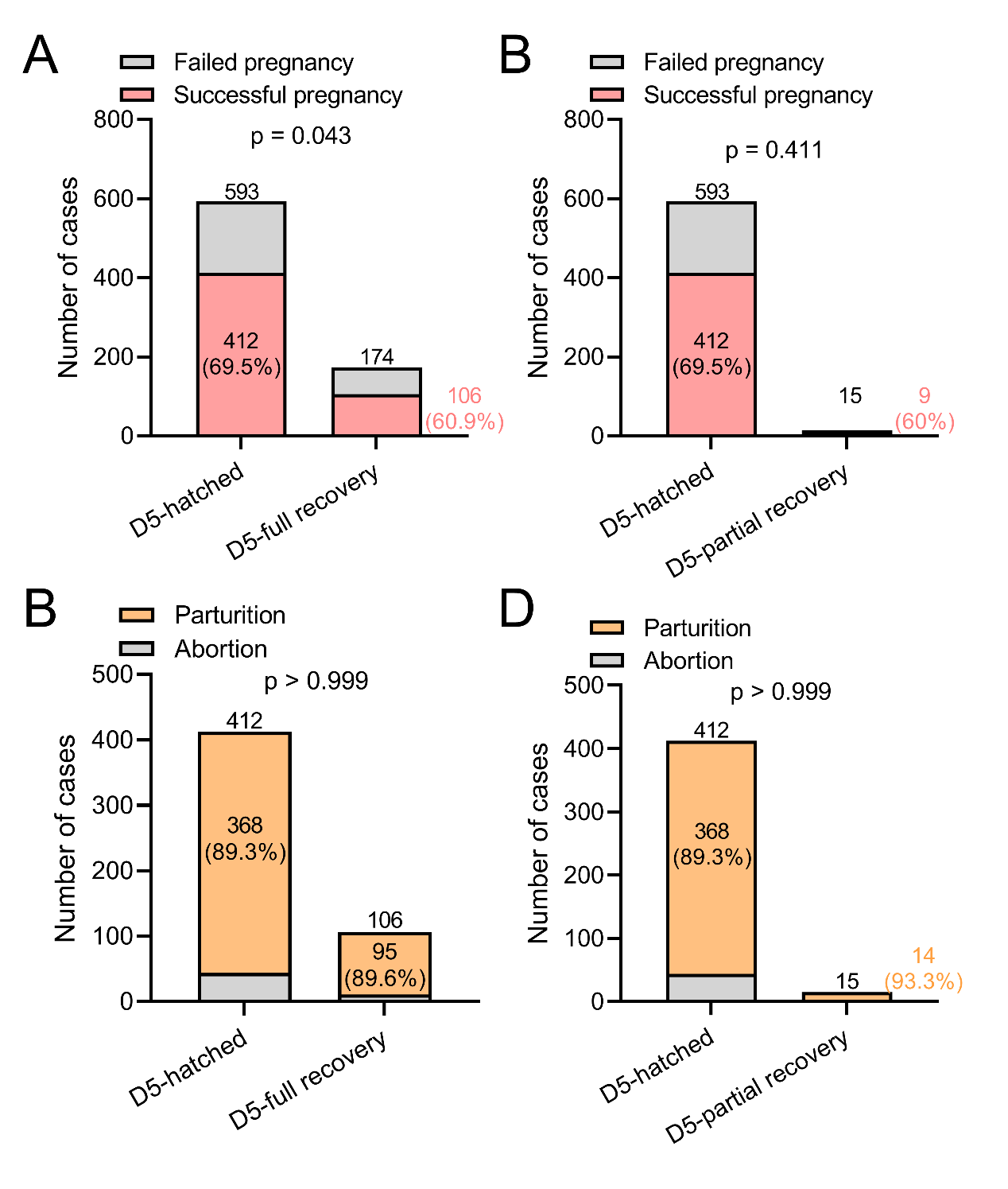


Figure S5. Comparisons of the pregnancy ratio (A-B) and abortion ratio (C-D) between patients received different incubation outcomes of blastocyst transplantation from frozen-thawed D5. Fisher’s exact test.
